# Supplementary material for: Behavior of Assembled Promyelocytic Leukemia Nuclear Bodies upon Asymmetric Division in Mouse Oocytes
Source: Int J Mol Sci. 2024 Aug 8;25(16):8656. doi: 10.3390/ijms25168656 (PMC11354524; doi:10.3390/ijms25168656)
Supplement: Supplementary file 1 [file ijms-25-08656-s001.zip › Supplemental Figure legends.pdf]

Supplemental figure legends

Figure S1, related to Fig. 1. Optimization of the assembly of PML-NBs in the nucleus of GV oocytes. Representative images of attempts for the exogenous assembly of PML-NBs in the nucleus of the GV oocyte. GV oocytes were injected with (A) 25 ng/µL EGFP-hPML VI mRNA (B) 150 ng/µL EGFP-hPML VI mRNA (C) 25 ng/µL hPML VI -sfGFP mRNA and cultured for (A) 27 hr (B) 21 hr (C) 24 hr, respectively. (A) Live cell images of before (upper panel) and after (lower panel, z-stack) the treatment of 6 µM arsenite (As) for 3 hr. (B) Live cell image. (C) Oocytes were fixed and stained with SUMO1 (red) antibody. BF, bright-field image of the oocyte. Scale bars, (A and B) 19.9 µm and (C) 20.0 µm, respectively.

Figure S2, related to Fig. 3. Assembled hPML VI -sfGFP mRNA-derived PML-NBs (hmdPML-NBs) after fertilization do not show an overt defect on the progression of development.

(A) Representative images of hmdPML-NBs assembled after insemination in the nucleus of the 2-cell embryo. Embryos were stained with anti-human PML (green) and anti-KAP1 (red) antibodies. For enlarged images of marked areas are shown in the insets. (B) Representative images of DPPA2 expression in the (B, upper) absence or (B, bottom) presence of hmdPML-NBs assembled after insemination. Embryos were stained with anti-human PML (green) and anti-DPPA2 (red) antibodies. Scale bars, (A) 19.9 and 2.00 (B, upper) 19.9 (B, bottom) 20.0 µm, respectively.

Figure S3, related to Fig. 4. The stalemate of hmdPML-NBs in the cytoplasm in the

prolonged stress condition.

(A) Representative images of the stalled hmdPML-NBs in the cytoplasm of metaphase oocytes at 40 hr. (B) Representative images of the hmdPML-NBs in the nucleus of arrested GV oocytes at 40 hr. Oocytes were stained with anti-human PML (green) antibody. Scale bars, 19.9µm.

Supplemental Video S1

CHO-K1 cells stably expressing GFP- PML VI (Hirano and Udagawa 2022) were employed in the time-lapse observation. Each of capture images was obtained every 2 minutes for 10 hours. Note rapid nucleation/appearance of PML-NBs in the nucleoplasm of daughter cells just after mitotic division.

Hirano, S., & Udagawa, O. (2022). Effects of arsenic on the topology and solubility of promyelocytic leukemia (PML)-nuclear bodies. *PloS one*, *17*(5), e0268835.
