# Supplementary figures and images for: Behavior of Assembled Promyelocytic Leukemia Nuclear Bodies upon Asymmetric Division in Mouse Oocytes
Source: Int J Mol Sci. 2024 Aug 8;25(16):8656. doi: 10.3390/ijms25168656 (PMC11354524; doi:10.3390/ijms25168656)

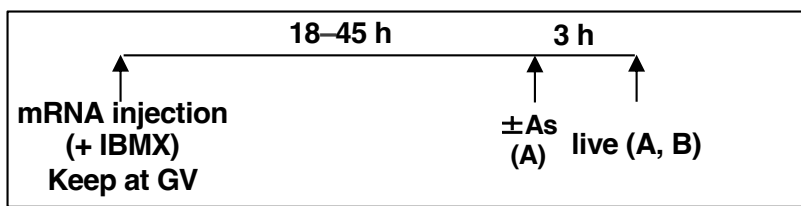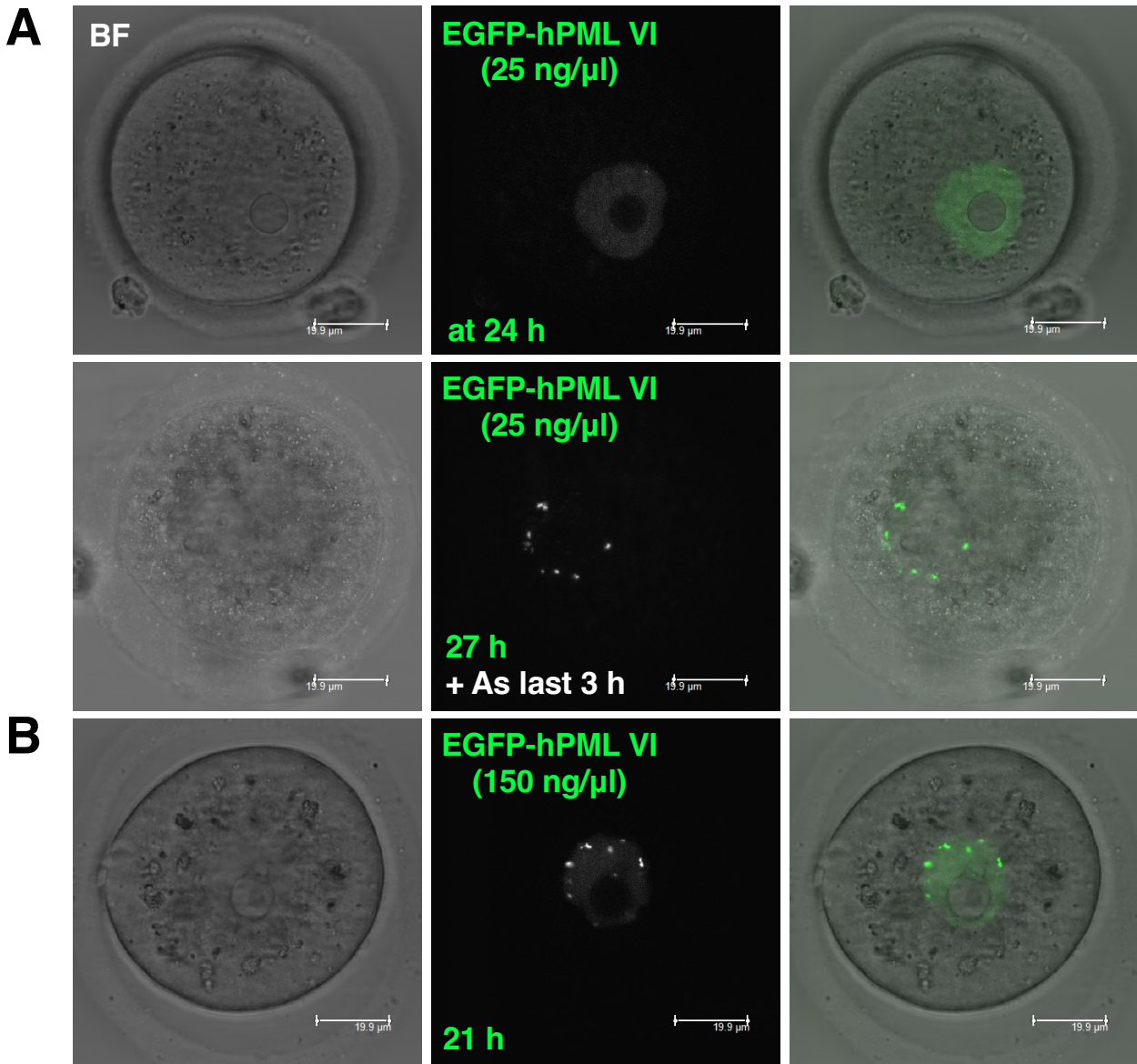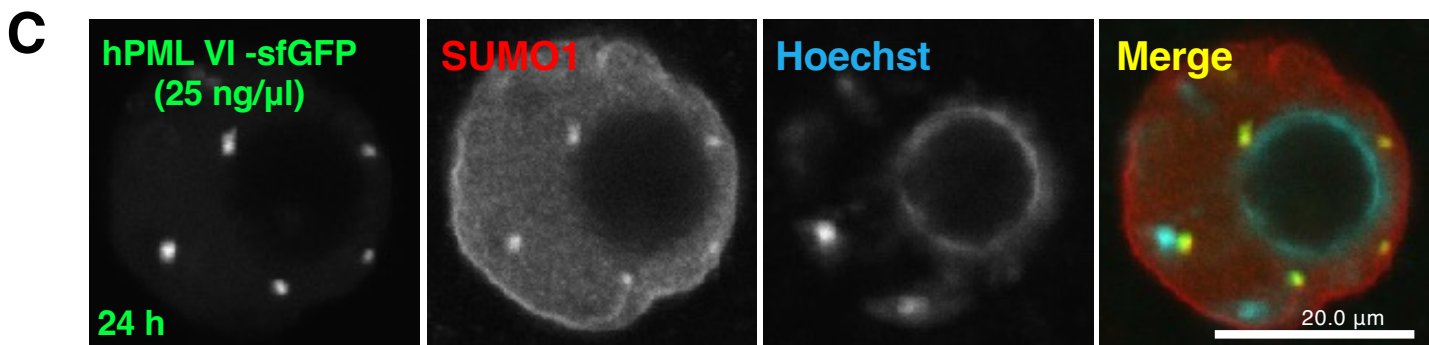

Supplement: Supplementary file 1 [file ijms-25-08656-s001.zip › Supplemental Figure S1.pdf]

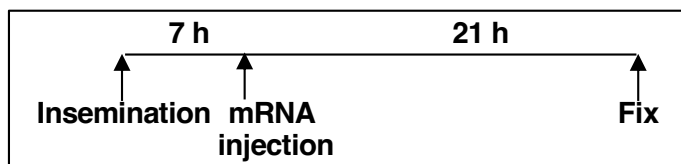

**A**

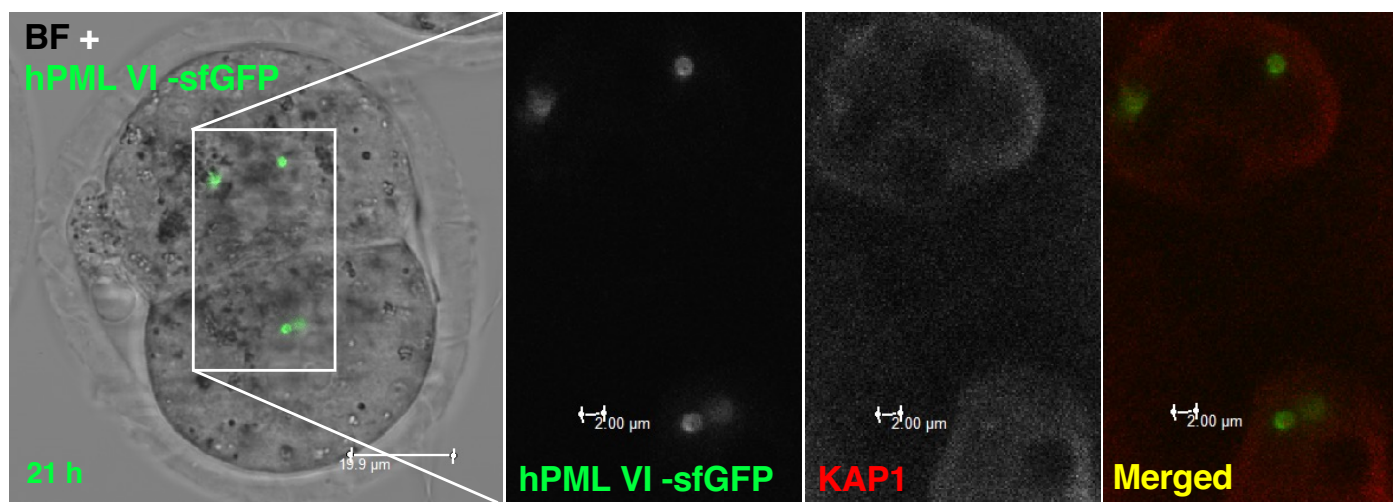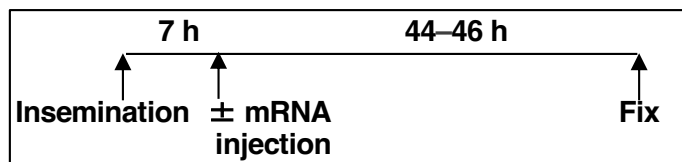

**B**

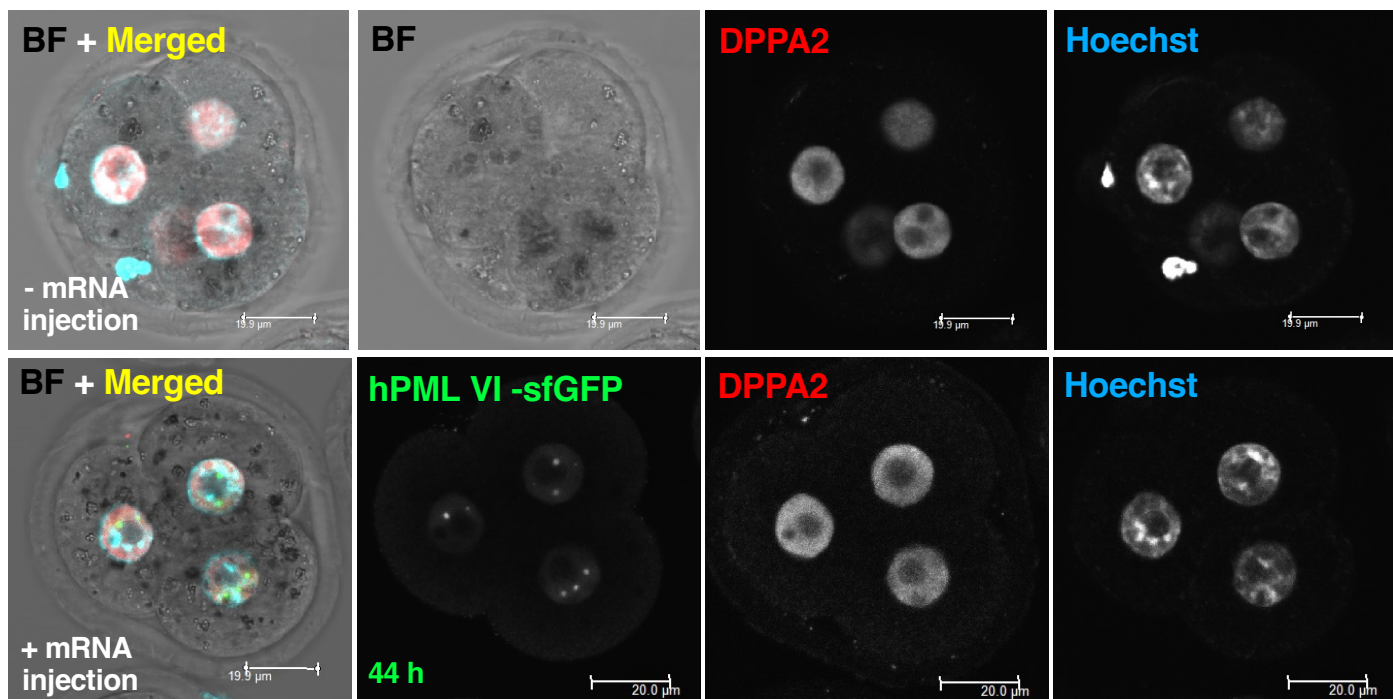

Supplement: Supplementary file 1 [file ijms-25-08656-s001.zip › Supplemental Figure S2.pdf]

**A****Metaphase Oocytes**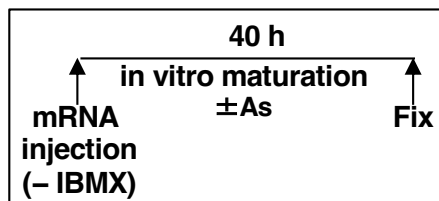**- As, ave. 24.0 NBs**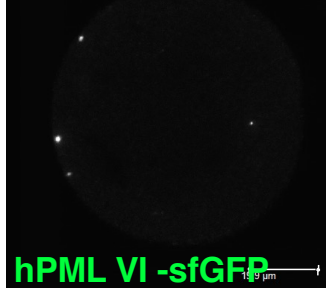**+ As, ave. 25.4 NBs**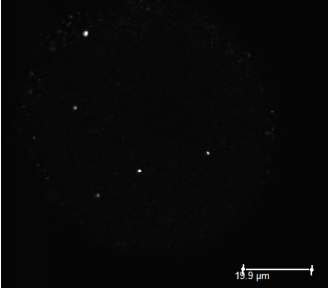**B****Arrested GV Oocytes****- As**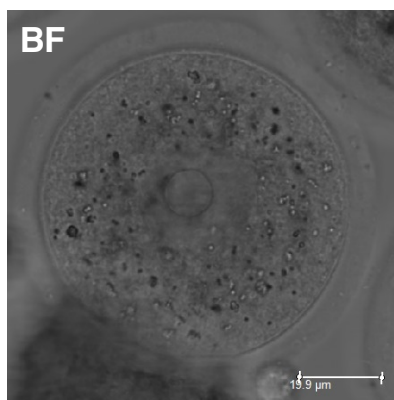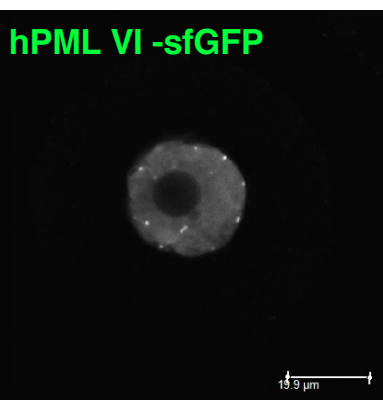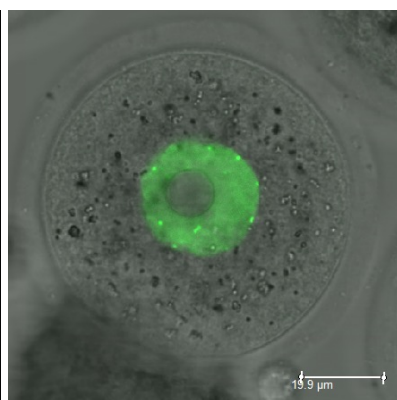**+ As**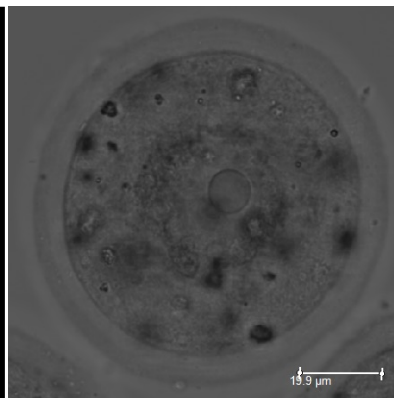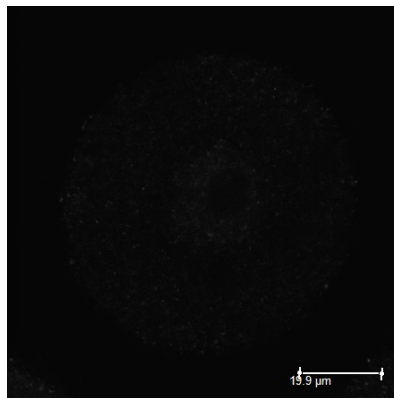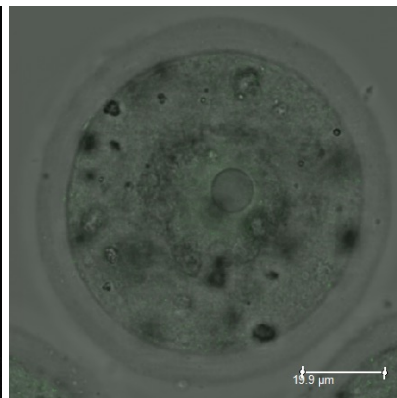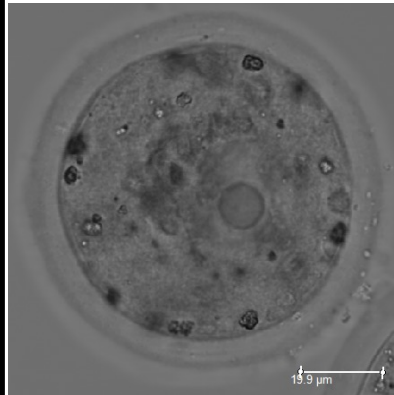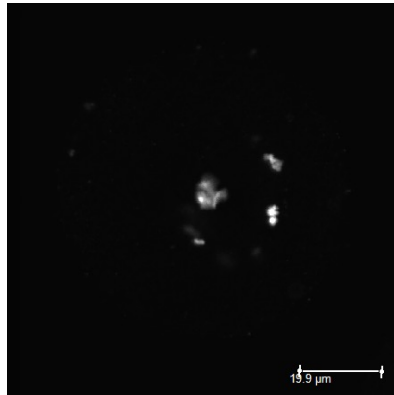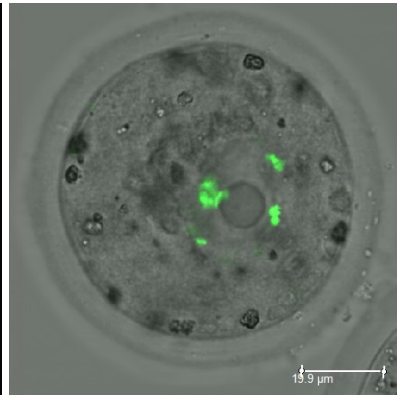

Supplement: Supplementary file 1 [file ijms-25-08656-s001.zip › Supplemental Figure S3.pdf]
